# Sugammadex bei Anaphylaxie durch Rocuronium

**DOI:** 10.1007/s00101-025-01549-y

**Published:** 2025-06-04

**Authors:** Silvia Schumacher, Martin Söhle, Florian Piekarski

**Affiliations:** Klinik für Anästhesiologie und Operative Intensivmedizin, Universitätsklinikum Bonn, Venusberg-Campus 1, 53127 Bonn, Deutschland

## Anamnese

Ein 64-jähriger männlicher Patient wurde zur geplanten roboterassistierten Prostatektomie bei azinärem Adenokarzinom der Prostata stationär aufgenommen. Die präoperative Evaluation gemäß American Society of Anesthesiologists (ASA) ergab leichte Allgemeinerkrankungen (ASA 2). Weitere relevante Begleiterkrankungen umfassten eine arterielle Hypertonie, chronische Zephalgien und eine Sinusitis. Frühere operative Eingriffe, darunter mehrere Leistenhernienreparationen sowie eine lumbale Spondylodese im Jahr 2021, waren ohne Komplikationen unter Allgemeinanästhesie durchgeführt worden.

## Anästhesie

Die Einleitung der Allgemeinanästhesie erfolgte mit Fentanyl, Lidocain, Propofol und Rocuronium. Etwa 30 s nach Applikation des Relaxans und während der problemlosen Maskenbeatmung kam es zu einer ausgeprägten Tachykardie. Eine zu geringe Narkosetiefe wurde zu diesem Zeitpunkt mittels kontinuierlicher EEG-Überwachung (Bispektralindex (BIS) = 37, Roh-EEG: dominanter Delta-Rhythmus) ausgeschlossen. Es folgte der Entschluss zur unmittelbaren endotrachealen Intubation. Nach konventioneller Laryngoskopie mittels Macintosh-Spatel wurde der Patient mit einem Endotrachealtubus der Größe 8.0 unter Sicht (Grad I gemäß der Cormack-Lehane-Klassifikation) problemlos intubiert. Nach manueller Beatmung zeigte sich kein endtidales CO_2_ in der Kapnographie, sodass die Tubuslage erneut mittels Laryngoskopie überprüft und bestätigt wurde. Bei weiterhin fehlendem endtidalen CO_2_ und erhöhten Beatmungsdrücken unter maschineller und manueller Beatmung wurde die Arbeitshypothese eines Bronchospasmus gestellt. Eine Vertiefung der Narkose zeigte jedoch zunächst nur gering zunehmende Atemvolumina bei weiter stark reduziertem endtidalem CO_2_. Kurz darauf zeigten sich eine ausgeprägte Hypotonie und nachfolgend ein generalisiertes Erythem, sodass die Diagnose des anaphylaktischen Schocks im Team gestellt wurde. Die initiale arterielle Blutgasanalyse (BGA) ergab eine respiratorische Acidose mit pH-Wert 7,21, pCO_2_ 46,8 mm Hg und einem Sauerstoffpartialdruck von 575 mm Hg unter kontrollierter Beatmung mit einer F_I_O_2_ von 1,0. Das EKG zeigte einen Sinusrhythmus ohne Zeichen einer Ischämie. Ein transthorakales Echokardiogramm (TTE) zeigte eine hyperdynamische linksventrikuläre Funktion bei Volumenmangel sowie eine regelrechte rechtsventrikuläre Funktion. Höhergradige Klappenvitien oder ein Perikarderguss wurden ausgeschlossen.

## Therapie und Verlauf

Nach der Diagnose eines anaphylaktischen Schocks wurde die Notfalltherapie mit intravenösem Adrenalin, ergänzt um kristalloide Infusionen, Adrenalin inhalativ, H_1_- und H_2_-Blocker sowie einem Kortikosteroid eingeleitet. Während sich die Beatmungssituation zeitnah normalisierte, konnte der Patient trotz der umfassenden Therapie, einschließlich Vasopressortherapie und großzügiger Volumengabe, zunächst nicht ausreichend hämodynamisch stabilisiert werden. Nach Diskussion im Anästhesieteam wurde die gezielte Antagonisierung des Rocuroniums durch Sugammadex als Ultima Ratio, um die immunologische Reaktion abzuschwächen, durchgeführt. Es konnte nun eine rasche und nachhaltige Stabilisierung der hämodynamischen Verhältnisse erreicht werden. Der Patient wurde anschließend unter stabilen respiratorischen und zirkulatorischen Bedingungen extubiert und auf die Intermediate-Care(IMC)-Station verlegt. Der Patient zeigte sich nach der Extubation wach, zu allen Qualitäten orientiert und klinisch stabil.

Im weiteren Verlauf wurde eine allergologische Diagnostik durch die Klinik für Dermatologie durchgeführt. Die Hauttestungen ergaben eine starke Sensibilisierung auf Rocuronium, während Lidocain, Propofol und Fentanyl keine Reaktionen auslösten. Als alternatives Muskelrelaxans wurde ferner Mivacurium getestet und im Verlauf problemlos verwendet. Der Patienten erhielt eine umfassende Aufklärung und einen Anästhesieausweis. Vier Wochen nach dem anaphylaktischen Schock wurde die robotisch assistierte Prostatektomie komplikationslos durchgeführt, wobei als Muskelrelaxans ausschließlich Mivacurium verwendet wurde.

## Diskussion

Anaphylaktische Reaktionen sind in der Anästhesiologie selten, stellen jedoch eine potenziell lebensbedrohliche Komplikation dar [1]. Die Inzidenz perioperativer Anaphylaxien der Grade 3–5 wird auf etwa 1:10.000 geschätzt, hierbei gehören neuromuskuläre Blocker wie Rocuronium und Succinylcholin zu den häufigen Auslösern [2]. Anaphylaktische Reaktionen können durch IgE-vermittelte Mechanismen ausgelöst werden; bei diesen führt die Exposition gegenüber einem Allergen wie Rocuronium zu einer Quervernetzung von IgE auf Mastzellen [3, 4]. Dies setzt Histamin, Tryptase und Prostaglandine frei, die zu den typischen Symptomen wie Bronchospasmus, Hypotonie, Vasodilatation und Hautreaktionen führen. Die Diagnose eines anaphylaktischen Schocks ist während der Allgemeinanästhesie herausfordernd, da die Symptome unspezifisch sein können und mit anderen Ursachen wie Volumenmangel oder Medikamentenreaktionen überlappen können. In diesem Fall wurden die Hypotonie, der Bronchospasmus und das generalisierte Erythem frühzeitig als Hinweise auf eine anaphylaktische Reaktion erkannt, was die sofortige Einleitung der Therapie ermöglichte.

Die AWMF-Leitlinie empfiehlt, dass bei fehlender Stabilisierung der Symptome und drohender Dekompensation von Atmung oder Kreislauf die i.v.-Applikation von Adrenalin erfolgen soll, obwohl sonst die i.m.-Gabe aufgrund geringerer kardiovaskulärer Nebenwirkungen bevorzugt wird [5]. Zusätzlich werden die Volumentherapie und die Gabe von H_1_-Antihistaminika empfohlen. Die Kombination aus H_1_- und H_2_-Blockern kann erwogen werden, jedoch ist die Evidenzlage für die Anwendung von H_2_-Blockern begrenzt. Die Gabe von Glukokortikoiden wird ebenfalls als nachrangig angesehen, da auch hier die Evidenz gering ist und insbesondere der Wirkungseintritt zeitverzögert erfolgt.

In diesem Notfall kam es nach der Gabe von Sugammadex zu einer raschen Stabilisierung der hämodynamischen Situation. Sugammadex bindet selektiv an Rocuronium und neutralisiert dessen Wirkung durch Enkapsulierung [6]. Zum Einsatz von Sugammadex bei der Therapie der durch Rocuronium bedingten Anaphylaxie existieren bislang nur Fallberichte und Fallserien [1]. Die Gabe von Sugammadex stellt in Fällen einer rocuroniuminduzierten Anaphylaxie eine wichtige Behandlungsoption dar, um die allergische Reaktion abzuschwächen und eine Stabilisierung des Patienten zu gewährleisten.

Die mediane Zeit bis zur vollständigen Reversierung von Rocuronium beträgt etwa 3 min [7]. Ein sofortiges Ansprechen auf Sugammadex im Rahmen der Anaphylaxietherapie wurde zwar in früheren Fallberichten beschrieben, jedoch als nicht durchgängig zuverlässig beurteilt [8]. Dieses Phänomen wird vermutlich durch die weiterhin frei liegende allergene Ammoniumgruppe erklärt; diese ist für die Bindung mit IgE und die daraus resultierende allergische Reaktion verantwortlich [6].

## Prävention

Nach der Stabilisierung des Patienten ist die Identifikation des auslösenden Agens essenziell, um zukünftige Ereignisse zu vermeiden. Hauttests, insbesondere der Prick- und Intradermaltest, sind dabei die diagnostischen Mittel der Wahl. In diesem Fall bestätigte die allergologische Diagnostik Rocuronium als auslösenden Faktor der Reaktion, während andere eingesetzte Medikamente wie Lidocain, Propofol und Fentanyl keine Sensibilisierung zeigten. Die allergologische Aufarbeitung ist für die Planung zukünftiger Eingriffe unerlässlich. Da intradermale Testungen mit Rocuronium bei Verdünnungen ≤ 1:1000 bereits falsch-positive Reaktionen auslösen können, sollte möglichst mit höheren Verdünnungen gearbeitet werden. Hintergrund hierfür ist das Auftreten positiver Hautreaktionen bei intradermalen Testungen von Rocuronium ohne Nachweis von Mastzelldegranulation, sofern die Verdünnung nicht eingehalten wird [9].

## Fazit für die Praxis

Dieser Fall unterstreicht die Bedeutung einer schnellen und zielgerichteten Therapie bei anaphylaktischen Reaktionen während der Narkose. Den höchsten Stellenwert nehmen bei der Behandlung schwerwiegender perioperativer Anaphylaxien die Gabe von Adrenalin und eine ausreichende Volumentherapie ein. Die Evidenzen für Antihistaminika und Kortison sind gering und sollten die Gabe von Adrenalin nicht verzögern. Sugammadex, als spezifischer Antagonist von Rocuronium, kann eine Behandlungsoption darstellen, insbesondere in therapierefraktären Fällen. Eine umfassende allergologische Diagnostik ist erforderlich, um zukünftige Risiken zu minimieren und geeignete alternative Medikamente, die bei zukünftigen Operationen verwendet werden können, zu identifizieren.
